# The Position of Charing Cross and King's College Hospitals

**Published:** 1895-06-15

**Authors:** 


### THE INSTITUTIONAL WORKSHOP.

### HOSPITAL ADMINISTRATION.

### THE POSITION OF CHARING CROSS AND KING'S COLLEGE HOSPITALS.

THE UNEQUAL DISTRIBUTION OF METROPOLITAN HOSPITALS.

AN urgent appeal issued by the president and treasurers of the Charing Cross Hospital, which appeared in the *Times* about three weeks ago, has led to a suggestion by correspondents in the same paper that Charing Cross Hospital should be closed, the site and existing buildings sold, and that the money should be devoted to the erection of a new hospital in Camberwell, to which the Charing Cross Hospital school should be transferred. The correspondents in question quoted the report of the Lords' Committee with evidence in favour of their suggestion, although a reference to it shows them to be in error. A good deal of evidence was given before the Lords' Committee on the unequal distribution of hospitals. It pointed out that, within a radius of a mile from Middlesex Hospital, there were twenty-six special and eight general hospitals, with an aggregate of about 2,050 beds, in addition to seven general and six special dispensaries and the Poor Law infirmaries. This and similar evidence, of which there was much, was fully weighed by the Lords' Committee, who found that great congestion of hospital accommodation did exist in certain metropolitan districts. Despite this, the Committee state* that "they cannot regard this suggestion to remove certain hospitals in places where they are not so much required to localities where the accommodation is deficient as practical." On the contrary, "they strongly advise that more hospital accommodation should be provided south of the Thames, where, say, in the densely populated district of Camberwell a large hospital would no doubt be of extreme value." It will thus be seen that, so far as the Lords' Committee are concerned, they condemn as unpractical the proposal made by the correspondents in the *Times* to transplant Charing Cross or any other hospital from its present site to the suburbs. It is well that this fact should be clearly grasped at the outset by those interested in the matter. On the other hand, special circumstances may render it desirable, as in the case of the old Great Northern Hospital, to remove the institution nearer the centre of the district to the needs of the population of which it ministers.

POINTS TO BE BORNE IN MIND.

At Islington in the beginning of 1882 a local committee, including several medical men, laboured assiduously to secure the establishment of a general hospital in a central position of that locality. At that time the Great Northern Hospital occupied several houses in the Caledonian Road, where the accommodation was inadequate and unsatisfactory in several respects. This latter fact caused the committee of the Great Northern Hospital to realise that they must rebuild the hospital on its existing or some other site. When the movement already referred to began in Islington the committee of the Great Northern Hospital expressed a wish to amalgamate the two schemes, a course which was adopted in the end. With much difficulty a site was secured, upon which a new and most complete modern hospital has been erected at a cost of about £100,000, and the new hospital is now known as the Great Northern Central. In this instance abundant proof is forthcoming of the wisdom of the policy pursued. The ordinary income of the old hospital in 1882 was £3,686. At the end of last year the income of the hospital amounted to £7,859. The invested funds in 1882 amounted altogether to £13,028; at the end of 1894 they were increased to £20,968, and after the sale of stock, realising upwards of £10,000, required to liquidate the building debt, the hospital retains invested funds which exceed £14,000. This success was undoubtedly due in the main to the genuine interest excited throughout the whole of North London in the new hospital, a circumstance which is proved by the fact that £15,000 was raised by the unaided exertions of the ladies of Islington. It must further be noted that the Great Northern Central Hospital was established upon new principles, for it combines the pay and free system of hospital relief, and has won its way to popular favour. This example points to the conclusion that to secure success it is essential that the whole population of the district in which the new hospital is to be built shall be favourable to the scheme, that from the commencement the principles upon which it is to be maintained shall commend themselves to public favour, and that the friends of any existing institution shall be induced to co-operate in the new scheme. Can these conditions be fulfilled in the case of King's College or Charing Cross Hospital and the Camberwell movement?

THE POSITION OF CHARING CROSS AND KING'S COLLEGE HOSPITALS.

King's College, like Charing Cross Hospital, has had to issue an urgent appeal for support within recent times. According to the latest figures available, it appears that the income obtained from invested property by King's College Hospital is £1,105 per annum, against £1,918 derived by Charing Cross Hospital from similar sources. King's College Hospital has an income of £2,267 from annual subscriptions, Charing Cross deriving an income of £1,908 from the same source. Charing Cross obtains double the income from nurses and probationers' fees that King's College Hospital does, the great difference between the pecuniary position of the two institutions being that King's College has established a five years' maintenance fund, whereas Charing Cross has not, and that the invested property of Charing Cross Hospital seems to be nearly double that of King's College Hospital. Turning next to the question of site, we find that King's College has 220 feet of site area per bed, compared with a site area per bed of only 86 feet at Charing Cross Hospital. On the other hand, the King's College site, or a portion of it, was once an old burial ground, a fact which accounts, no doubt, for some, if not all, the unhealthiness which has been accredited to King's College Hospital in the past. In the matter of buildings, the advantage undoubtedly

* Paragraph 588 of their Report.

lies with Charing Cross Hospital, as neither the lavatories and bath-rooms nor the wards and offices at King's College Hospital are in accordance with modern requirements and hygienic completeness. Again, the site of Charing Cross Hospital is excellent, so far as the needs of the neighbourhood in which it is situated are concerned. It stands on one portion of a triangular space, all buildings upon which not at present under the control of the hospital authorities, might no doubt be purchased, and the whole of the land added to the present site. If this were to be done Charing Cross Hospital might be made one of the most perfect of metropolitan hospitals. It seems to us, therefore, if the best interests of the population of each of these two hospitals are to be taken into consideration on their merits, that if one of them is to be closed, it would be far better to close King's College than Charing Cross Hospital. If this view prevail, as we think it ought to, unless both hospitals are to continue on their present basis, there need be no difficulty in amalgamating the two schools and making the combined hospital building on the enlarged site where Charing Cross Hospital now stands a splendid example of modern hospital construction and efficiency.

### The Crucial Test.

We are disposed to think the correspondent of the *Times* has done an ill service to Charing Cross Hospital by raising this question at the moment when the authorities were doing their best to procure funds to carry on the useful work they undoubtedly do for London. We have purposely refrained from expressing a definite opinion on the present occasion, because we wished to ascertain whether the suggestion of the *Times*' correspondent was made with the sanction of the governing body of Charing Cross Hospital, and what their views are upon this question. We should also like to hear what the authorities of King's College Hospital have to say in the matter. Again, supposing it be determined to close either Charing Cross or King's College Hospital, and to transfer the staff to a new hospital at Camberwell, what prospect is there of funds being available from the sale of the site of either institution, and the realisation of its funds, having regard to the fact that the amalgamation of the two existing medical schools, and the necessity of making the old hospital financially secure, must absorb the whole or at any rate a greater portion of the money so realised? These are matters for the gravest consideration, and until they are settled it is idle to talk of transplanting one or both institutions from its present site to a new one in south-east London or elsewhere.

